# CBCT assessment of crestal bone changes and stability: Immediate versus delayed implant placement in grafted sites

**DOI:** 10.6026/973206300220776

**Published:** 2026-02-28

**Authors:** Mounika Prashanthi, Aella Balakrishna, Kouser Anees, Mohammad Wasimuddin, Seethal, Asim Mustafa Khan, Md Kafeel Ahmed

**Affiliations:** 1 Department of Oral Medicine and Radiology, G Pulla Reddy Dental College and Hospital, Kurnool, Andhra Pradesh, India; 2 Department of Oral and Maxillofacial Surgery, Sri Sai College of Dental Surgery, Vikarabad, Telangana, India; 3 Department of Prosthodontics and Crown and Bridge, Late YCMM and RDF's Dental College and Hospital, Ahilyanagar, Maharashtra, India; 4 Department of Oral Medicine and Radiology, Consultant in Calicut, Kerala, India; 5 Department of Biomedical Dental Sciences, College of Dentistry, Imam Abdulrahman Bin Faisal University, Dammam, Saudi Arabia; 6 Department of Periodontology and Implantology, MNR Dental College and Hospital, Sangareddy, Hyderabad, Telangana, India

**Keywords:** Dental implants, immediate implant placement, delayed implant placement, cone-beam computed tomography (CBCT), crestal bone loss, implant stability, bone grafting

## Abstract

Alveolar ridge resorption after tooth extraction complicates optimal implant positioning and increases grafting requirements. Therefore, it is of interest to compare immediate versus delayed implant placement in 60 grafted extraction sites using Cone-beam computed tomography (CBCT) and resonance frequency analysis. Delayed placement preserved more buccal bone (-0.42±0.28 mm loss) than immediate placement (-0.89±0.34 mm, p<0.001), with comparable mesial/distal changes. Both achieved equivalent implant stability, integration and survival after 12 months. Delayed placement advances socket management by optimizing buccal contour preservation without compromising outcomes, informing timing decisions.

## Background:

This phenomenon has already become a proven treatment modality as the endosseous dental implants utilized to replace the missing teeth have predicted long-term results and high satisfaction rates among the patients [1]. The time of the realization of the implant after extracting the teeth is, however, a controversial issue in clinical practice and different guidelines prove their unique benefits and shortcomings based on site-specifics and individual patient features [2]. To maximize the clinical outcomes, it is important to understand the biological mechanisms that regulate post-extraction alveolar healing and how they can be applied to planning the treatment of the implant. After tooth extraction, there is marked alteration in the dimensional structure of the alveolar ridge with a pronounced bone resorption of the alveolar ridge and consequential remodelling of the socket walls [3]. Since the buccal bone plate is generally thinner than the lingual/palatal one, landmark studies by Aaraujo and Lindhe revealed more advanced vertical and horizontal resorption in the former which may lead to the undermining of future placement of implants and the aesthetic results [4]. The changes in dimension are the fastest during the 3-6 months after extraction, with the average horizontal dimension reduction of 29-63% and the vertical dimension reduction of 11-22% described in systematic studies [5]. Immediate implant placement which is characterized by the insertion of implants after extracting the tooth was introduced to potentially avoid such dimensional changes as well as to minimize the overall treatment duration and the number of surgical procedures [6]. Advocates of this system indicate that socket architecture maintenance by inserting the implant immediately can be used to retain peri-implant bone sizes and improve the appearance of soft tissues [7]. Nevertheless, other studies have shown that placement does not help to avoid physiological remodelling of the buccal bone plate (at least in the short run) in the presence of thin biotypes [8]. On the other hand, delayed implant placement protocols, which are usually done 4-6 months after extraction, enable full recovery of the soft tissue and partial bone before the insertion of the implants [9]. Such a technique will allow a more predictable control of the flaps, evaluation of the healed ridge morphology and allow bone augmentation at the same time when necessary [10]. Opponents of delayed protocols give reasons such as longer time to heal, possibility of further resorption of the ridge during the healing period and high morbidity among patients due to the repeated surgeries [11]. Extraction bone grafting has become a technique used to reduce the dimensional change post-extraction and create the best environment in which the next implant is to be placed irrespective of timing protocol [12].

Preservation of socket with different biomaterials such as xenografts, allografts and synthetic substitutes have been shown to be effective in terms of preserving the ridge size when compared with natural healing [13]. The grafting with immediate or delayed implant protocols is the combination that is used in modern clinical practice, but there are not very many comparative data on long-term outcomes. Cone-beam computed tomography Cone-beam computed tomography (CBCT) has transformed the evaluation of changes in peri-implant bone and currently allows the assessment of the crestal bone of a tooth within three dimensional evaluations at a resolution of less than one millimetre [14]. In contrast to two-dimensional radiography, CBCT allows measuring the buccal and lingual/palatal bone dimensions which would have been hidden by the presence of implant superimposition [15]. Also, resonance frequency analysis (RFA) offers objective assessment of implant stability, by measuring the stiffness of the implant-bone interface, in terms of the values of the implant stability quotient (ISQ). Although much has been written about implant timing protocols, few comparative studies have been done to specifically compare the immediate and delayed placement of implants in grafted sites utilizing extensive three-dimensional assessment. Besides, the interconnection between timing protocol, crestal bone maintenance and the formation of the implication stability of augmented sites needs additional clarification [16]. These relationships are also vital to the evidence-based treatment planning and patient counselling. Therefore, it is of interest to compare the crestal bone level at insertion and implant stability of immediate and delayed implantation placement protocols in grafted extraction sites using both the CBCT imaging and resonance frequency analysis within 12 months of post loading protocol follow up.

## Materials and Methods:

## Study design:

This prospective comparative clinical study was conducted at the Department of Oral Implantology, University Dental Hospital, between January 2021 and March 2024.

## Sample size calculation:

Sample size estimation was performed using power analysis based on previous literature reporting mean buccal crestal bone loss of 1.2±0.5 mm with immediate placement and 0.7±0.4 mm with delayed placement protocols. Assuming α=0.05, power of 80% and anticipated effect size of 0.75, a minimum of 26 patients per group was required. Accounting for potential 15% dropout rate, 30 patients were enrolled in each group, totaling 60 participants.

## Patient selection:

Patients were recruited from the implant clinic based on predefined eligibility criteria.

## Inclusion criteria:

[1] Adults aged 18-65 years

[2] Presence of single tooth requiring extraction in the maxillary or mandibular premolar/molar region

[3] Adequate bone volume for placement of standard-diameter implant (≥3.75 mm diameter, ≥10 mm length) following grafting

[4] Indication for bone grafting due to anticipated ridge deficiency

[5] Good general health without uncontrolled systemic conditions

[6] Commitment to follow-up attendance for the study duration

[7] Non-smoker or light smoker (≤10 cigarettes/day)

## Exclusion criteria:

[1] Acute periapical infection or abscess at extraction site

[2] Uncontrolled diabetes mellitus (HbA1c >7.5%)

[3] History of bisphosphonate therapy or radiation to head/neck region

[4] Immunocompromised status or immunosuppressive medication

[5] Severe parafunctional habits (bruxism)

[6] Pregnancy or lactation

[7] Inability to maintain adequate oral hygiene (full-mouth plaque score >25%)

[8] Extraction sites requiring extensive vertical augmentation (>5 mm)

## Group allocation:

Patients were allocated to two groups based on treatment timing preference following informed discussion of both protocols:

## Group A (Immediate Placement):

Implant placement performed immediately following atraumatic tooth extraction with simultaneous bone grafting (n=30)

## Group B (Delayed Placement):

Socket preservation grafting at extraction, followed by implant placement after 4 months of healing (n=30)

## Surgical protocol:

All surgical procedures were performed by a single experienced implantologist under local anesthesia (4% articaine with 1:100,000 epinephrine). Atraumatic extraction was accomplished using periotomes and extraction forceps without flap elevation. Following extraction, sockets were thoroughly debrided and curetted to remove granulation tissue.

## Immediate placement protocol (Group A):

Following extraction, implant osteotomy was prepared in the palatal/lingual aspect of the socket according to prosthetically-driven positioning. Tapered implants (Straumann BLT, Institut Straumann AG, Basel, Switzerland) were placed with primary stability ≥25 Ncm. The gap between the implant surface and buccal bone plate was grafted with deproteinized bovine bone mineral (Bio-Oss, Geistlich Pharma AG, Wolhusen, Switzerland) and covered with a resorbable collagen membrane (Bio-Gide, Geistlich Pharma AG). Primary wound closure was achieved with tension-free suturing using 5-0 polyglycolic acid sutures.

## Delayed placement protocol (Group B):

Extraction sockets were filled with deproteinized bovine bone mineral and covered with collagen membrane. Primary closure was achieved and sites were allowed to heal for 4 months. At the second surgical stage, a crestal incision with minimal flap reflection was performed and implants were placed following standard osteotomy protocol. Additional grafting was performed if residual defects were present. All patients received standardized postoperative instructions, including chlorhexidine 0.12% mouthrinse twice daily for 2 weeks, amoxicillin 500 mg three times daily for 7 days and ibuprofen 400 mg as needed for pain management.

## Implant loading and prosthetic protocol:

Implants were loaded after 4 months of submerged healing in both groups. Second-stage surgery involved punch technique or minimal flap for healing abutment placement. Final cement-retained porcelain-fused-to-metal crowns were delivered 2 weeks following second-stage surgery.

## CBCT imaging protocol:

CBCT scans (Carestream CS 9300, Carestream Dental, Atlanta, GA, USA) were obtained at three time points:

[1] T0: Baseline (immediately post-surgery for Group A; immediately post-implant placement for Group B)

[2] T1: 4 months post-loading

[3] T2: 12 months post-loading

Imaging parameters included 90 kVp, 4 mA, voxel size 0.2 mm and field of view 8x8 cm. Images were reconstructed perpendicular to the implant long axis for standardized measurements.

## Radiographic measurements:

The levels of crestal bone were quantified between the implant platform and the initial bone-implant contact at four sites, that is, mesial, distal, buccal and palatal/lingual. Two blind examiners who were not aware of group allocation were used to make measurements with specific software (CS 3D Imaging, Carestream Dental). Change in crestal bone level was determined by the difference between the follow-up and baseline measurements where negative values reflected bone loss.

## Implant stability evaluation:

The resonance frequency analysis (Osstell ISQ, Osstell AB, and Gothenburg, Sweden) was used to determine the level of implant stability during placement, 4 months (loading), 8 months and 12 months after loading. The measurements were taken in mesio-distal and bucco-palatal directions and the average of the ISQ was taken.

## Outcome variables:

## Primary outcome:

The change in the level of crestal bone at mesial, distal, buccal and palatal/lingual positions at 12 months after loading.

## Secondary outcomes:

Stabilization of the implant (IMD values) at given intervals, survive rate of the implant and complications.

## Statistical analysis:

The SPSS version 26.0 (IBM Corporation, Armonk, NY, USA) was used to analyze the data. Continuous variables were indicated in terms of the mean standard deviation and the categorical variables in terms of frequencies and percentages. Shapiro-Wilk test was used to test normalcy. Between-group comparisons made were done using independent samples t-test when sample data follows a normal distribution and Mann-Whitney U test when the data is non-parametric. Paired t -test or Wilcoxon signed-rank test were used to analyse within-group changes. Categorical variables were compared using Chi-square test. Inter-examiner reliability with radiographic measurements was determined with the help of the intraclass correlation coefficient (ICC). The level of statistical significance was at p<0.05.

## Results:

Sixty patients were enrolled and completed the study protocol, with no dropouts during the 12-month follow-up period. Baseline demographic and clinical characteristics demonstrated comparable distribution between groups (Table 1). Mean age was 47.3±11.2 years in Group A and 45.8±12.6 years in Group B (p=0.632). Gender distribution, smoking status, implant location and bone quality showed no significant differences. Mean implant dimensions were similar between groups, with all implants achieving adequate primary stability for immediate loading consideration. Inter-examiner reliability for CBCT measurements demonstrated excellent agreement, with ICC values ranging from 0.91 to 0.96 for all measurement sites. Crestal bone level changes at 4 months and 12 months post-loading are presented in Table 2. At the buccal aspect, Group A (immediate placement) demonstrated significantly greater bone loss compared to Group B (delayed placement) at both 4 months (-0.68±0.31 mm versus -0.31±0.24 mm, p<0.001) and 12 months (-0.89±0.34 mm versus -0.42±0.28 mm, p<0.001). The palatal/ lingual aspect showed similar trends, with greater bone loss in Group A at 12 months (-0.52±0.29 mm versus -0.34±0.22 mm, p=0.008). Mesial and distal crestal bone changes were comparable between groups at both time points. At 12 months, mesial bone loss was -0.38±0.26 mm in Group A versus -0.35±0.23 mm in Group B (p=0.634), while distal bone loss was -0.41±0.28 mm versus -0.37±0.25 mm (p=0.561). Within-group analysis revealed significant bone loss from baseline to 12 months at all sites in both groups (p<0.001 for all comparisons). ISQ values demonstrated characteristic stability dip patterns in both groups during the healing phase (Table 3). At implant placement, mean ISQ values were 67.2±5.8 in Group A and 69.4±6.2 in Group B (p=0.159). At loading (4 months post-placement), ISQ values were 71.8±4.6 and 72.4±5.3, respectively (p=0.637). Final ISQ values at 12 months post-loading were comparable between groups (74.8±4.2 versus 73.6±5.1, p=0.312). Both groups demonstrated significant increases in ISQ from baseline to 12 months post-loading (p<0.001), indicating successful osseointegration progression. Implant survival rate was 100% in both groups at 12-month follow-up, with no implant failures recorded. Minor complications occurred in 4 patients (13.3%) in Group A (2 cases of transient wound dehiscence, 2 cases of membrane exposure) compared to 2 patients (6.7%) in Group B (1 case of wound dehiscence, 1 case of localized infection managed with antibiotics). The difference in complication rates was not statistically significant (p=0.389).

## Discussion:

This is a prospective controlled trial with a three-dimensional evidence of crestal bone remodelling and the compliance of implants in immediate and delayed implant placements in grafted extraction sites. Our results have shown that a delay in placement of 4 months following socket healing led to substantially better retention of buccal crestal bone and the same long-term retention of implants as standard protocols of immediate placement. The much higher loss of the buccal bone with immediate placement (-0.89±0.34 mm versus -0.42±0.28 mm at 12 months) is consistent with the biological principles of post-extraction remodelling [17]. The buccal bone plate that is mainly made of bundle bone when relying on periodontal ligament vasculature suffers unavoidable resorption after tooth extraction, even when implants are immediately inserted [18]. Our results are in line with the results of Suaid *et al.* who established that immediate implant placement does not inhibit the physiological remodelling of the buccal wall [19]. Even though the grafting material offers osteoconductive scaffold, it is not able to entirely offset biological impulse of the buccal plate resorption in the face of a combined surgical trauma and implant placement. On the contrary, the delayed protocol of placing allowed extraction socket healing and partial regeneration in advance before implant insertion to provide a more stable biological milieu in which the process of osseointegration takes place. Buser *et al.* suggested an implant timing classification system that acknowledges the advantages of permitting the soft tissue to develop prior to the placement of the implant especially in aesthetically challenging scenarios [20]. This rationale is supported in the discovery of less bone loss in the buccal area when placed later which indicates that the period of 4 months allows the grafted socket to become solid enough to resist further surgery. Interestingly, the mesial and distal crestal bone alterations were similar across groups, which is probably due to the stronger bone structure at the interproximal locations and the existence of the adjacent tooth root support that sustains the bundle bone viability [21]. Such site-specific remodelling pattern has significant clinical implications on treatment planning, especially in anterior areas where the buccal bone mass has been shown to be the most important in supporting the soft tissue and esthetic efficiency. The measurements of the implant stability showed some typical patterns of the initial stability of the implants in both groups and steadily growing to the stability of biological integration [22]. Although there was a difference in the preservation of the buccal bones, the final values of ISQ were similar between the groups showing that both the timing protocols result in successful results of the osseointegration. This result is consistent with systematic reviews that showed similar survival rates of implants placed immediately and delayedly under the condition of proper case selection and surgical practice used [23]. Nevertheless, the larger change of ISQ in the immediate placement group (+7.6 as compared to +4.2) is likely to be due to the lack of initial stability of fresh extraction sockets, which require more significant bone development throughout the healing process. The overall CBCT analysis used in the present study has specific benefits over the traditional radiographic techniques in the analysis of the bone changes around the implant [24]. Although two-dimensional periapical radiographs can be effectively used in monitoring the interproximal bone levels, they do not provide any accurate representation of the buccal and palatal/ lingual bone dimensions owing to the implant superimposition. It was found during our three-dimensional analysis that the major variations in the bone remodeling patterns would have been overlooked with the standard imaging methods and the importance of CBCT in conducting the research and multifaceted clinical examination became clear. To isolate the effect of time of implant placement, the grafting protocol that used xenograft material and collagen membrane cover was standardized in the two groups. Deproteinized bovine bone mineral has shown to have positive osteoconductive characteristics, as well as, low rates of resorption, which offer long lasting volume retention [25]. Consistent with prior clinico-radiographical evidence, immediate implant placement demonstrates greater buccal crestal bone loss compared to delayed protocols, despite comparable overall survival rates [26]. The similarity in the graft material volume and method between groups augers well with the soundness of our comparisons in terms of time. The clinical applicability of our findings may depend on a number of factors. One, the study population mainly consisted of premolar sites and molar sites, the aesthetic requirements might not be as high as in anterior areas. The extrapolation to anterior maxillary positions should be done carefully since the thin buccal bone phenotype that is usually present anteriorly will enhance the differences, which are being studied in this paper [27]. Second, the 4-month healing period chosen to be used in delayed placement is a trade-off between permitting sufficient socket healing and the reduction in treatment time; other durations of healing can be used with different results. The increased incidence of complication which is found with immediate placement (13.3% versus 6.7) though not significant is worth considering. Although normally manageable, wound dehiscence and membrane exposure may undermine graft integration and aesthetic results [28]. The single-step surgical method, in which the extraction, the placing of the implant and the grafting processes are performed simultaneously, is always more complex and prone to complications than the staged ones. The immediate placement protocol has several benefits that benefit the patient such as shorter treatment and fewer surgical procedures, which can be considered as patient-centered. Nevertheless, these advantages have to be considered on the background of the increased crestal bone remodelling, especially at the buccal side. The protocol choice should be based on shared decision-making taking into consideration the preferences of the patient, aesthetic considerations and site-specific anatomical factors. The weakness of this study is connected with the non-randomized allocation method, the use of a single center and 12 months follow-up. More protracted research is required to determine whether these bone level differences seen at 12 months are maintained or stabilize with time. Also, soft tissue aesthetic, patient-reported quality measurement and cost-effectiveness analysis would be useful additional information to assist in clinical decision-making. Further studies are necessary to determine whether certain patient or site factors can be used to anticipate varying responses to timing protocols to allow personalization of the treatment planning. The possible contribution of adjunctive treatment, such as growth factors and alternative grafting materials to optimize the results in various timing conditions also deserves research.

## Conclusion:

We show that delaying implant placement by four months after socket preservation leads to significantly better buccal crestal bone preservations about 50% less loss while maintaining similar long-term implant stability. Both immediate and delayed protocols achieved 100% implant survival, with differences mainly observed in buccal bone remodelling rather than mesial or distal aspects. Delayed placement is preferable in cases with compromised buccal bone or high aesthetic demands, whereas immediate placement remains viable for adequate bone volume and time-sensitive treatments.

## Figures and Tables

**Table 1:** Baseline patient and implant characteristics

**Variable**	**Group A: Immediate (n=30)**	**Group B: Delayed (n=30)**	**p-value**
Age, years (Mean±SD)	47.3±11.2	45.8±12.6	0.632
Gender, n (%)			0.795
- Male	14 (46.7)	13 (43.3)	
- Female	16 (53.3)	17 (56.7)	
Smoking status, n (%)			0.688
- Non-smoker	24 (80.0)	22 (73.3)	
- Light smoker	6 (20.0)	8 (26.7)	
Implant location, n (%)			0.542
- Maxillary premolar	12 (40.0)	10 (33.3)	
- Maxillary molar	8 (26.7)	11 (36.7)	
- Mandibular premolar	6 (20.0)	4 (13.3)	
- Mandibular molar	4 (13.3)	5 (16.7)	
Implant diameter, mm (Mean±SD)	4.1±0.3	4.2±0.4	0.286
Implant length, mm (Mean±SD)	10.8±1.2	11.1±1.0	0.298
Primary stability, Ncm (Mean±SD)	32.4±6.8	35.2±7.4	0.138
Initial ISQ (Mean±SD)	67.2±5.8	69.4±6.2	0.159
Baseline buccal bone thickness, mm	1.2±0.4	1.4±0.5	0.097

**Table 2:** Crestal bone level changes (mm) at follow-up time points

**Measurement Site**	**Time Point**	**Group A: Immediate (n=30)**	**Group B: Delayed (n=30)**	**p-value**
**Mesial**				
	T1 (4 months)	-0.28±0.21	-0.25±0.19	0.567
	T2 (12 months)	-0.38±0.26	-0.35±0.23	0.634
**Distal**				
	T1 (4 months)	-0.31±0.24	-0.27±0.21	0.494
	T2 (12 months)	-0.41±0.28	-0.37±0.25	0.561
**Buccal**				
	T1 (4 months)	-0.68±0.31	-0.31±0.24	<0.001*
	T2 (12 months)	-0.89±0.34	-0.42±0.28	<0.001*
**Palatal/Lingual**				
	T1 (4 months)	-0.39±0.25	-0.26±0.20	0.028*
	T2 (12 months)	-0.52±0.29	-0.34±0.22	0.008*
*Statistically significant (p<0.05);
Negative values indicate
bone loss from baseline

**Table 3:** Implant Stability Quotient (ISQ) values over time

**Time Point**	**Group A: Immediate (n=30)**	**Group B: Delayed (n=30)**	**p-value**
Implant placement	67.2±5.8	69.4±6.2	0.159
4 months (loading)	71.8±4.6	72.4±5.3	0.637
8 months post-loading	73.4±4.4	72.8±4.9	0.616
12 months post-loading	74.8±4.2	73.6±5.1	0.312
Change (baseline to 12 months)	+7.6±3.2	+4.2±2.8	<0.001*
*Statistically significant (p<0.05)

## References

[1] Wang Y (2021). BMC Oral Health..

[2] Peitsinis PR (2025). J Clin Med..

[3] Salem CQ (2023). Natl J Maxillofac Surg..

[4] Araújo MG, Lindhe J (2005). J Clin Periodontol..

[5] Sah N (2025). Cureus..

[6] Canullo L (2022). Clin Oral Investig..

[7] Atri F, Nokar K (2024). Clin Exp Dent Res..

[8] Staas TA (2022). Clin Implant Dent Relat Res..

[9] Mishra A (2022). Int J Clin Pediatr Dent..

[10] https://pocketdentistry.com/.

[11] Chau KK (2025). J Dent..

[12] Fu WT (2024). J Dent Res..

[13] Kurt-Bayrakdar S (2025). Dentomaxillofac Radiol..

[14] Siqueira R (2021). Clin Oral Implants Res..

[15] Chappuis V (2013). J Dent Res..

[16] Muthaiyan V (2025). J Indian Soc Periodontol..

[17] ElNahass H (2024). Clin Implant Dent Relat Res..

[18] Bungthong W (2022). Molecules..

[19] Suaid FA (2014). Clin Oral Implants Res..

[20] Buser D (2017). Periodontol 2000..

[21] Verma V (2022). J Oral Biol Craniofac Res..

[22] Srinivasan R (2025). Natl J Maxillofac Surg..

[23] Patel R (2023). Dent J (Basel)..

[24] Song D (2021). BMC Med Imaging..

[25] Sánchez-Pérez A (2025). Biomedicines..

[26] Shitole AM (2022). Int J Med Biomed Stud..

[27] Chen K (2021). J Prosthodont..

[28] Garcia J (2018). Clin Oral Implants Res..

